# Early post-approval experience of the selective cytopheretic device surveillance registry for pediatric AKI requiring kidney replacement therapy

**DOI:** 10.1007/s00467-026-07181-1

**Published:** 2026-02-06

**Authors:** Stuart L. Goldstein, Kelli A. Krallman, Meredith Harris, Rajit K. Basu, David J. Askenazi, Shina Menon, Matt S. Zinter, Stephen M. Gorga, Harvey David Humes, Emily C. Scribe, David Austin Catanzaro, Sai Prasad N. Iyer, Kevin K. Chung

**Affiliations:** 1 University of Cincinnati College of Medicine, Cincinnati Children’s Hospital Medical Center, 3333 Burnet Avenue, Cincinnati, OH 45229 USA; 2 Lurie Children’s Hospital, Chicago, IL USA; 3 University of Alabama at Birmingham, Birmingham, AL USA; 4 Division of Nephrology, Department of Pediatrics, Stanford University, Palo Alto, CA USA; 5 University of California at San Francisco, San Francisco, CA USA; 6 University of Michigan, Ann Arbor, MI USA; 7 SeaStar Medical, Denver, CO USA

**Keywords:** SCD, Selective cytopheretic device, Immunomodulation, Acute kidney injury, Kidney replacement therapy, Sepsis

## Abstract

**Background:**

The selective cytopheretic device for pediatrics (SCD-PED) was granted a Humanitarian Device Exemption (HDE) in 2024 by United States Food and Drug Administration (FDA) for treatment of critically ill children weighing ≥ 10 kg with AKI due to sepsis or a septic condition on antibiotics and requiring continuous kidney replacement therapy (CKRT). FDA required a post-approval surveillance registry to collect additional safety data among all patients treated with SCD-PED under the HDE as part of the approval.

**Methods:**

The *S*CD-PED Pedi*A*tric Sur*V*eillance R*E*gistry (SAVE; NCT06517810) was initially planned to enroll 300 patients under the HDE-approved indication, but the target was later reduced to 50. The primary outcome is a new (secondary) blood stream infection in the first 28 days after SCD-PED initiation. Additional Day 28 and Day 90 clinical outcomes including mortality and KRT requirement will be collected.

**Results:**

Registry design and preliminary outcomes from the first 21 patients are reported. Three new positive blood cultures were observed during the follow-up period after cessation of SCD-PED therapy, all of which were deemed unrelated to SCD. Sixteen (76%) and 15 (71%) of 21 patients survived through to Days 60 and 90, respectively. Three patients died while receiving SCD-PED therapy. No device-related adverse events were reported.

**Conclusion:**

This initial real-world experience of SCD-PED therapy in pediatric AKI with sepsis in patients requiring CKRT suggests no device-related infections and similar improved clinical outcomes consistent with the previous trial SCD-PED experience in this critically ill population.

**Graphical abstract:**

**Figure:**
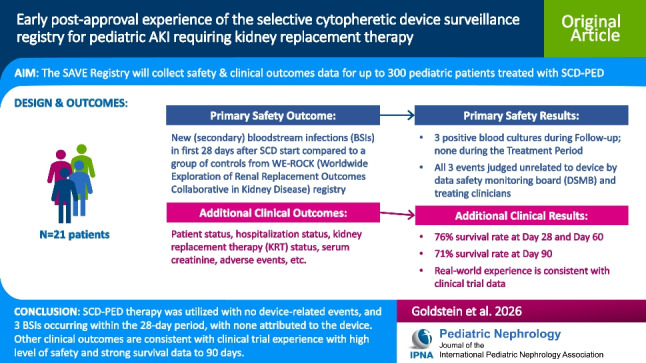
A higher resolution version of the Graphical abstract is available as Supplementary information

**Supplementary information:**

The online version contains supplementary material available at 10.1007/s00467-026-07181-1.

## Background

Acute kidney injury (AKI) requiring continuous kidney replacement therapy (CKRT) is a serious and often life-threatening condition in pediatric patients, particularly those with concomitant sepsis or a septic-like condition. Sepsis-associated AKI is characterized by a dysregulated immune response, systemic inflammation, and endothelial dysfunction, all of which contribute to the historically poor outcomes observed in this population [1]. Current therapeutic strategies for managing pediatric sepsis-associated AKI remain limited and are supportive in nature, necessitating innovative approaches to enhance patient survival [2].

A multicenter study from the prospective pediatric CKRT (ppCRRT) registry of 116 children with multiorgan dysfunction (MOD) receiving CKRT, including 47 (39%) with sepsis, showed that ICU survival was 51.7% [3]. A recent single-center study of children receiving CKRT revealed a similar survival rate in 130 patients receiving a vasoactive medication (50.0%) or mechanical ventilation (50.7%) on CKRT [4]. A recent multicenter (32 centers, 7 countries) study from the Worldwide Exploration of Renal Replacement Outcomes Collaborative in Kidney Diseases (WE-ROCK) of 980 patients (birth—25 years of age) who received CKRT for AKI or pathologic fluid accumulation reported 64.1% survival to ICU discharge [5, 6]. A secondary analysis from the same cohort showed that patients with sepsis at CKRT initiation were less likely to liberate from CKRT by 28 days (30% vs. 38%; *p* < 0.001) and had higher mortality rates (47% vs. 31%; *p* < 0.001) compared to non-septic patients [7].

The selective cytopheretic device for pediatrics (SCD-PED) represents a potential breakthrough in the management of patients with sepsis-associated AKI requiring CKRT. The SCD-PED is a novel cell-directed immunomodulatory therapy that is connected in series to a commercially available CKRT circuit, and in the presence of regional citrate anticoagulation, it modulates inflammation by targeting pro-inflammatory neutrophils and monocytes to shift them to a lower inflammatory phenotype [8]. These immunomodulatory effects of the SCD have been well-documented both in vitro and in vivo. Various studies have demonstrated that the SCD selectively binds the more activated circulating neutrophils and monocytes. Only a small percentage (3–5%) of the circulating population of activated neutrophils and monocytes are bound, so the SCD is not immunosuppressive in its effect. Previous work demonstrates that bound neutrophils in the low ionized environment of blood within the SCD promotes apoptosis and returns these cells into circulation [8–10]. In addition, recent data utilizing single-cell RNAseq analysis suggest that the SCD effectively reprograms circulating monocytes from a pro-inflammatory to an anti-inflammatory, reparative phenotype [11]. These cell processing events result in a tempering of the systemic inflammatory response as reflected clinically with reductions in blood levels of multiple pro-inflammatory cytokines in various hyperinflammatory states [9, 10, 12, 13]. Importantly, this immunomodulation is achieved in a non-depletive manner, in contrast to cytokine-absorbing filters (e.g., CytoSorb™ or Oxiris™) that decrease cytokines by removing them directly from the vascular space.

Pooled analysis from two prospective multicenter studies (*n* = 22 children with AKI requiring CKRT and MOD weighing ≥ 10 kg) demonstrated a 77% survival rate, with no device-related serious adverse events observed [14]. Importantly, none of the survivors were dialysis-dependent at 60 days, and 87.5% had recovered normal kidney function. Based on these findings, the SCD-PED (QUELIMMUNE™, SeaStar Medical, Denver, CO) received Humanitarian Device Exemption (HDE) approval from United States Food and Drug Administration for use in critically ill children ≥ 10 kg with AKI accompanied by sepsis or septic conditions receiving antibiotics and requiring CKRT. As part of this approval, FDA required a post-approval surveillance registry to collect additional safety data prospectively among all patients treated with the device under the HDE. We report on the study design of the *S*CD-PED Pedi*A*tric Sur*V*eillance R*E*gistry, (SAVE Registry) and preliminary results from the first 21 patients treated post-approval, including those in SAVE.

## Methods

**Fig. 1:**
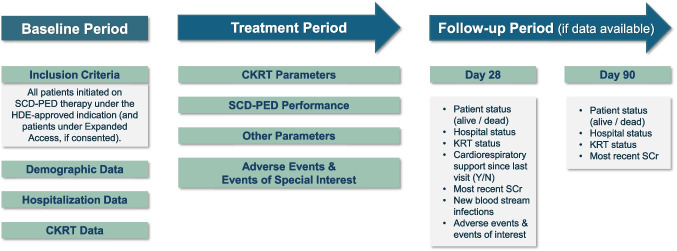
SAVE registry design overview

The SAVE Registry (ClinicalTrials.gov identifier: NCT06517810; Primary IRB: Cincinnati Children’s Hospital: 2024–0153; 25-April-2024) is a mandatory, prospective, post-approval surveillance registry with a primary objective to collect descriptive safety data on the clinical use of the SCD-PED under the HDE. We anticipate that approximately 50 participating institutions in the USA will enroll up to 300 patients in the registry. During review of this manuscript, this target was subsequently reduced to 50 by the FDA [15]. Each patient treated with SCD-PED therapy according to its FDA-approved indication under the HDE will be enrolled in the registry. All enrolled patients will receive the standard of care ICU treatment for those undergoing CKRT. There are no specific exclusion criteria for the registry. Additionally, patients treated with the SCD-PED under Expanded Access will be included in the registry, if informed consent is obtained. Informed consent/assent for the registry is mandatory prior to therapy. The treatment period (see Fig. 1) begins when the patient starts SCD-PED therapy and will continue until therapy completion. Therapy completion is defined as > 72 h without re-initiation of SCD-PED therapy. Follow-up will continue out to 90 days.

The SCD protocol has been described previously [14]. The SCD cartridge and tubing is integrated into the CKRT circuit post-CKRT membrane and changed at least every 24 h. All centers provide CKRT with regional citrate anticoagulation as part of their local standard of clinical care with the following Instructions for Use (IFU) required constraints: (1) regional citrate anticoagulation to maintain CKRT circuit iCa^2+^  < 0.40 mmol/L for at least 90% of the time and a continuous intravenous calcium infusion to maintain patient systemic iCa^2+^  > 1.0 mmol/L, and (2) only polysulfone-based CKRT membranes could be used (i.e., no polyacrylonitrile membranes). In contrast to the SCD-PED clinical studies, where the treatment duration was limited to ≤ 10 days, the treatment duration will be determined by the clinical team and can exceed 10 days if the patient continues to receive CKRT as part of the clinical standard of care. SCD discontinuation occurs at the discretion of the clinical team.

**Fig. 2:**
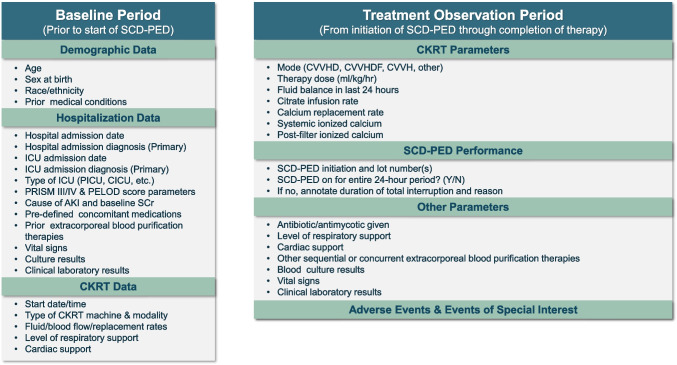
Data collection

At baseline, demographic data, hospitalization data, and CKRT data will be collected (see Fig. 2). During the treatment period, CKRT parameters, SCD-PED performance, adverse events of special interest, and other parameters will be collected.

The primary outcome is a new (secondary) blood stream infection (BSI) in the first 28 days after SCD-PED initiation or through hospital discharge, whichever is sooner. A matched cohort of CKRT patients with sepsis who did not receive treatment with SCD-PED from the WE-ROCK registry [5] will serve as controls. Additional outcomes (see Fig. 2) collected Day 28 include patient survival status, hospitalization status, kidney replacement therapy status, cardiorespiratory support, most recent serum creatinine, adverse events, and events of interest. An additional follow-up at Day 90 is being performed to match the WE-ROCK dataset for patient status, hospitalization status, kidney replacement therapy status, and most recent serum creatinine.

An independent data and safety monitoring board (DSMB), composed of two clinicians and a biostatistician, will regularly review safety data throughout the registry in accordance with FDA guidance. The board will assess adverse events, bloodstream infections, and deaths, meeting at least quarterly and ad hoc as needed. To support objective oversight, the DSMB operates under a formal charter and has already established data review protocols.

Initial post-approval outcomes analysis included patients enrolled in SAVE, as well as patients treated under emergency use post-approval who could not be included in the registry.

## Results

The SAVE Surveillance Registry was initiated in July 2024, and preliminary outcomes data are available from 20 patients across five registry sites. Of the 20 patients, two patients were treated under emergency use due to not meeting the HDE indication criteria. One patient exceeded the upper age limit of 22 years, and the other had a body weight below 10 kg. Following treatment, both individuals were subsequently included in the registry to facilitate the collection of safety and outcome data. One additional patient was treated under emergency use at a sixth site and was recently published as a case report [16] but could not be included in SAVE due to lack of informed consent for the registry. However, since this episode occurred after HDE approval, data from this case are included for this analysis, resulting in a total of 21 patients available for early outcome analysis. Treated patients were critically ill with AKI or acute kidney worsening requiring CKRT, had sepsis, and a variety of severe underlying illnesses with significant complexities, including those with malignancies, history of kidney transplant and/or kidney failure, chronic immunosuppression or immunodeficiencies, and open wounds (see Supplementary Table 1 for individual patient characteristics and hospital/ICU diagnoses). Unless otherwise noted, all metrics refer to the cohort of 21 patients. Median age at ICU admission was 9.2 years (interquartile range [IQR]: 5.3,16.8; range 1.1–23.7; *n* = 21), and median weight was 21.5 kg (IQR: 16.5, 45.1; range 9–105; *n* = 17) (Table 1). The cohort was 47% White, 19% Hispanic, 10% Black, 5% Asian/Pacific Islander, with 19% of other/unknown background. Median PRISM III score at ICU admission was 15 (IQR: 8, 22; range 0–31). At the time of SCD-PED initiation, median PELOD-2 score was 9 (IQR: 6, 11; range 3–15), and all patients were receiving vasoactive medications and mechanical ventilation. Nearly 20% of patients (4/21) had a history of kidney failure and/or kidney transplant and were KRT-dependent prior to treatment (Supplementary Table 2), and 33% (7/21) had receipt of extracorporeal membrane oxygenation (ECMO) either concomitantly (*n* = 5) with or immediately prior (*n* = 2) to treatment with SCD-PED. The median number of SCD treatment courses was 1 (IQR: 1, 1; range 1–3), and the median total duration of therapy (DoT) was 5 days (IQR: 2,10; range 1–33). The median days from CKRT to SCD-PED initiation was 2 (IQR: 0, 4; range 0, 97), with six patients initiated within a 24-h period (median of 4.5 h [IQR: 0, 15.35; range 0, 19.8]). The SCD-PED devices were switched out every 24 h per protocol throughout the treatment course.

**Table 1:** Patient demographics and baseline details

Category (*N* = # of patients with data reported)	Results
Patients enrolled	21
Median (IQR) age, years (*N* = 21)	9.2 (5.3, 16.8)
Median (IQR) weight, kg (*N* = 17)	21.5 (16.5, 45.1)
Median (IQR) height, cm (*N* = 17)	113 (99.8, 152.4)
Sex (male/female) (*N* = 21)	11/10
Median (IQR) PRISM III score at ICU admission (*N* = 19)	15 (8, 22)
Median (IQR) PELOD-2 score at SCD-PED initiation (*N* = 20)	9 (6, 11)
Receipt of vasoactive medications (%) (*N* = 21)	21 (100)
Intensive mechanical ventilation (%) (*N* = 21)	21 (100)
*Received extracorporeal membrane oxygenation* (*N* = 19): Concurrently with SCD-PED, *n* (%) Prior to SCD-PED, *n* (%)	5 (25) 2 (10)
History of kidney failure and/or recent kidney transplant prior to treatment (%)	4 (20)
Median (IQR) days from ICU to CKRT (*N* = 21)	4 (0, 18)
Median (IQR) days from CKRT to SCD-PED start (*N *= 21)	2 (0, 4)
Median (IQR) SCD-PED treatment days (per course) (*N* = 21)	5 (2, 10)
Median (IQR) SCD-PED treatment course (*N* = 21)	1 (1,1)

*CKRT* continuous kidney replacement therapy,  *ICU* intensive care unit, *IQR* interquartile range, *PELOD-2* pediatric logistic organ dysfunction-2 score, *PRISM III* pediatric risk of mortality score, *SCD-PED* selective cytopheretic device for pediatrics

Outcomes data were available for all 21 patients at the time of this analysis (Table 2). Three patients had positive blood cultures at 6, 7, and 22 days after the termination of SCD-PED therapy. One culture was positive (ampicillin-resistant *Klebsiella*) for ~ 32 h and was negative the following day, while the other two cultures were positive for *Enterococcus* and *Enterobacter*. All infections were deemed unrelated to SCD-PED by the treating clinician and the DSMB (see Supplementary Table 4 for additional details). Additionally, there were no device-related adverse events reported, including no reports of immunosuppressive effects (e.g., leukopenia or neutropenia). Sixteen (76%) of 21 patients survived through the treatment period, Day 28, and Day 60. Three patients died while receiving SCD-PED therapy. Fifteen (71%) of 21 patients survived through Day 90. Three of five patients concomitantly treated with SCD-PED and ECMO survived to Day 90. Among the survivors who were not KRT-dependent prior to treatment, 9 out of 12 (75%) and 10 out of 12 (83%) patients were KRT-independent at Day 28 and Day 90, respectively. Individual Day 28, 60, 90, and KRT patient outcomes are listed in Supplementary Table 3.

**Table 2:** Preliminary results

Category	Results		
Patients enrolled	21		
Device-related events	0		
*Positive blood cultures:* During treatment period During follow-up period (28 days post-initiation) Deemed as related to SCD-PED by DSMB and PI	0 3 0		
*Survival*: Day 28, *n* (%) Day 60, *n* (%) Day 90, *n* (%)	*All patients* 16/21 (76) 16/21 (76) 15/21 (71)	*ECMO only*^†^ 3/5 (60) 3/5 (60) 3/5 (60)	*CKD 5/Tx*^‡^ 4/4 (100) 4/4 (100) 3/4 (75)
*KRT independence among survivors*: Day 28, *n* (%) Day 90, *n* (%)	9/12 (75) 10/12 (83)		

^†^Five patients concomitantly treated with SCD-PED and ECMO, ^‡^four patients were KRT-dependent prior to SCD-PED therapy; of these, four patients at Day 28 and three at Day 90 were excluded from the KRT outcome as they were KRT-dependent prior to SCD-PED treatment

*DSMB* data safety monitoring board, *ECMO* extracorporeal membrane oxygenation, *CKD 5* Chronic Kidney Disease stage 5, *Hx* medical history, *PI* principal investigator, *KRT *renal replacement therapy, *SCD-PED* selective cytopheretic device for pediatrics, *Tx* kidney transplant

## Discussion

Pediatric AKI requiring CKRT in the setting of sepsis and/or MOD continues to result in significant morbidity and mortality. Here, we report the first real-world clinical experience of SCD-PED therapy in this critically ill pediatric population including patients from the FDA-mandated SAVE registry as part of the HDE. The HDE approval of SCD-PED (QUELIMMUNE™) for pediatric sepsis-associated AKI requiring CKRT was based on safety and probable benefit demonstrated in a cohort of 22 patients. Because the approval of SCD-PED was predicated on a relatively small patient population, FDA mandated the implementation of a post-market registry to monitor the safety of the device, especially as it relates to secondary bloodstream infections. Regarding the choice of BSI as the primary outcome, this reflects the focus on patient safety and the potential risks associated with extracorporeal devices in critically ill patients. While survival is an important secondary outcome, infection risk is considered a more immediate and clinically significant safety concern for this vulnerable patient population. Indeed, a recent publication by Quickfall [17] demonstrated that nearly 60% of ICU patients receiving CKRT had at least one positive culture, with intra-abdominal and BSI having the highest mortality (~64% and ~58%, respectively). The registry was therefore designed to prioritize monitoring for infections as part of its core safety objectives. As mentioned above, three BSIs occurred within the 28-day period post-SCD-PED initiation, with none attributed to the device. Given that one culture was only positive for ~32 h and the other two are generally considered to be common nosocomial infections (albeit associated with high mortality rates) [17], the lack of any unusual infections is reassuring and consistent with similar lack of any device-related infections from the SCD-PED-01 and −02 registrational studies. Furthermore, there was no evidence of immunosuppression, in the form of opportunistic bacterial, viral, or fungal infections, consistent with previous similar safety analyses from prior SCD clinical studies in patients with AKI requiring CKRT and a recent analysis of the effect of SCD treatment on neutrophil to lymphocyte ratios (NLR) from prior AKI studies [18, 19]. Definitive conclusions will depend on the pre-specified comparison with a matched cohort from the WE-ROCK study, which will be conducted upon completion of the full enrollment target of 300 patients (subsequently reduced to 50) in the SAVE registry. Accordingly, the current safety data from these initial 21 patients should be regarded as preliminary and descriptive.

Notably, many of these patients treated with SCD-PED therapy were critically ill, with nearly 1/4 receiving concomitant ECMO and 100% receiving vasoactive medications and invasive mechanical ventilation. The median PRISM III score at ICU admission was 15 with a range of complexities (PRISM III range of 0 to 31) and suggested patients with potentially higher severity than the SCD-PED clinical studies (PRISM II of 9.5) [14]. Importantly, almost half these children would not have been eligible for enrollment in the SCD-PED registrational clinical trials due to the severity of their illness or concomitant conditions, or to immune deficiencies or chronic immunosuppression (one patient had SLE and one patient had neutropenic sepsis [16]) (Supplementary Table 1). SCD-PED clinical use patterns were generally broad and ranged from early post-CKRT initiation starts to more advanced settings including salvage. In some cases, CKRT was initiated immediately upon admission to the ICU, followed with SCD-PED therapy within 24–48 h (Supplementary Table 1; three patients were concurrently initiated on CKRT and SCD-PED therapy). Most patients only received one SCD-PED treatment course, with a median duration of 5 days. The treatment duration was like the registrational study duration of 6 days [14]. Importantly, patient survival rates were 76% at Day 28 and Day 60 with 71% still alive at Day 90, which is like those observed in the registrational studies and higher than the historical ppCRRT cohort of 50%. Ten out of 12 patients who survived to Day 90 were KRT-independent. The two patients who were still KRT-dependent had complex chronic multi-organ inflammatory disease, with persistent pancreatitis and gastrointestinal perforation. Thus, they represented outliers, requiring at least four-fold greater SCD-PED therapy duration than the remainder of the cohort (23 and 36 days, respectively vs*.* cohort median of 5 days) (Supplementary Table 1). As such, they would have been excluded from the original clinical trials. It is important to note that SCD-PED therapy was terminated for these two patients as they achieved clinical stability and did not require CKRT and were able to transition to intermittent hemodialysis.

The findings from this initial cohort, including registry patients, demonstrate results consistent with the clinical trial experience that led to the initial HDE approval of the SCD, where a 77% 60-day survival was observed in pooled analysis from the SCD-PED-01 and SCD-PED-02 studies in patients with AKI requiring CKRT [14]. This post-approval cohort of 21 patients nearly doubles the overall pediatric experience with the SCD-PED to 43 patients. Furthermore, as prior reports on the SCD were limited to 60-day outcomes, this report is the first to present 90-day outcomes in either pediatric or adult patients with AKI requiring CKRT, especially within the context of real-world clinical evidence. We continue to interpret our outcome data with caution, as these data are primarily from an observational registry, so no efficacy claims can be made at this time. Yet, a 76% 60-day and 71% 90-day survival outcome in real-world clinical experience is generally favorable when considering the illness severity of the population, their co-morbid conditions, and the complexities of their treatment courses. Despite the small numbers (3 out of 5), the 60% survival outcome in ECMO patients is also promising, given that none of the ECMO patients from the registrational SCD-PED-01 and −02 studies survived [14]. This early cohort data now further expands evidence of SCD-PED in ECMO patients with AKI requiring CKRT. Similarly, a 75% survival in the four patients who were KRT-dependent prior to treatment with SCD-PED at the Day 90 timepoint now provides evidence of the SCD’s potential utility in an unexplored subgroup of pediatric patients. Most importantly, no device-related adverse events or any other safety signals were reported with SCD-PED therapy, especially when considering that the early cohort patients were generally sicker and considerably more complex than the patients from the SCD-PED clinical studies [7].

We acknowledge several limitations to the current study. First, the outcomes described here are from a small set of patients, the majority of whom were from an observational registry and thus are subject to limitations typical of such sources, including data incompleteness. For example, certain clinical components of the PRISM III (such as Glasgow Coma Scale) and PELOD-2 indices were not available in some patients, resulting in a possible underestimation of their severity and organ status. Finally, the survival and KRT dependence outcomes presented are non-comparative and can only be considered as preliminary and descriptive.

In summary, the first real-world experience of SCD-PED therapy in pediatric hospitals across the USA demonstrates results consistent with the clinical trial experience in both safety and survival rates. We plan on performing comprehensive comparative outcomes analysis against a matched cohort from WE-ROCK upon completion of the enrollment target of the SAVE registry. While the WE-ROCK cohort is more contemporary than the ppCRRT, it may be subject to era-specific characteristics that can impact direct comparisons. Yet, we could only provide a direct comparison by randomizing eligible patients with CKRT, with vs. without SCD therapy. Given the recognized high mortality rate of the eligible pediatric population, and the strong safety signal with increased evidence for potential direct benefit of the SCD, we suggest there will be little equipoise in the pediatric critical care nephrology community to commit to patient randomization. However, the ongoing adult trial is a prospective randomized trial of CKRT-SCD vs*.* CKRT alone [20], which will hopefully prove positive and provide the gold standard comparison.

## Supplementary information

Below is the link to the electronic supplementary material.

Graphical abstract (PPTX 86.5 KB)

ESM 2 (DOCX 41.5 KB)

ESM 3 (DOCX 24.0 KB)

ESM 4 (DOCX 36.4 KB)

ESM 5 (DOCX 23.9 KB)

## Data Availability

The data are available in the Supplementary Tables where the reader can refer to them. The demographic data are in Supplementary Table 1, and the survival outcomes are in Supplementary Table 3.
