# Supplementary material for: Early post-approval experience of the selective cytopheretic device surveillance registry for pediatric AKI requiring kidney replacement therapy
Source: Pediatr Nephrol. 2026 Feb 6;41(7):2205–12. doi: 10.1007/s00467-026-07181-1 (PMC13197363; doi:10.1007/s00467-026-07181-1)
Supplement: Supplementary file 1 — Graphical abstract (PPTX 86.5 KB) [file 467_2026_7181_MOESM1_ESM.pptx]

## Slide 1
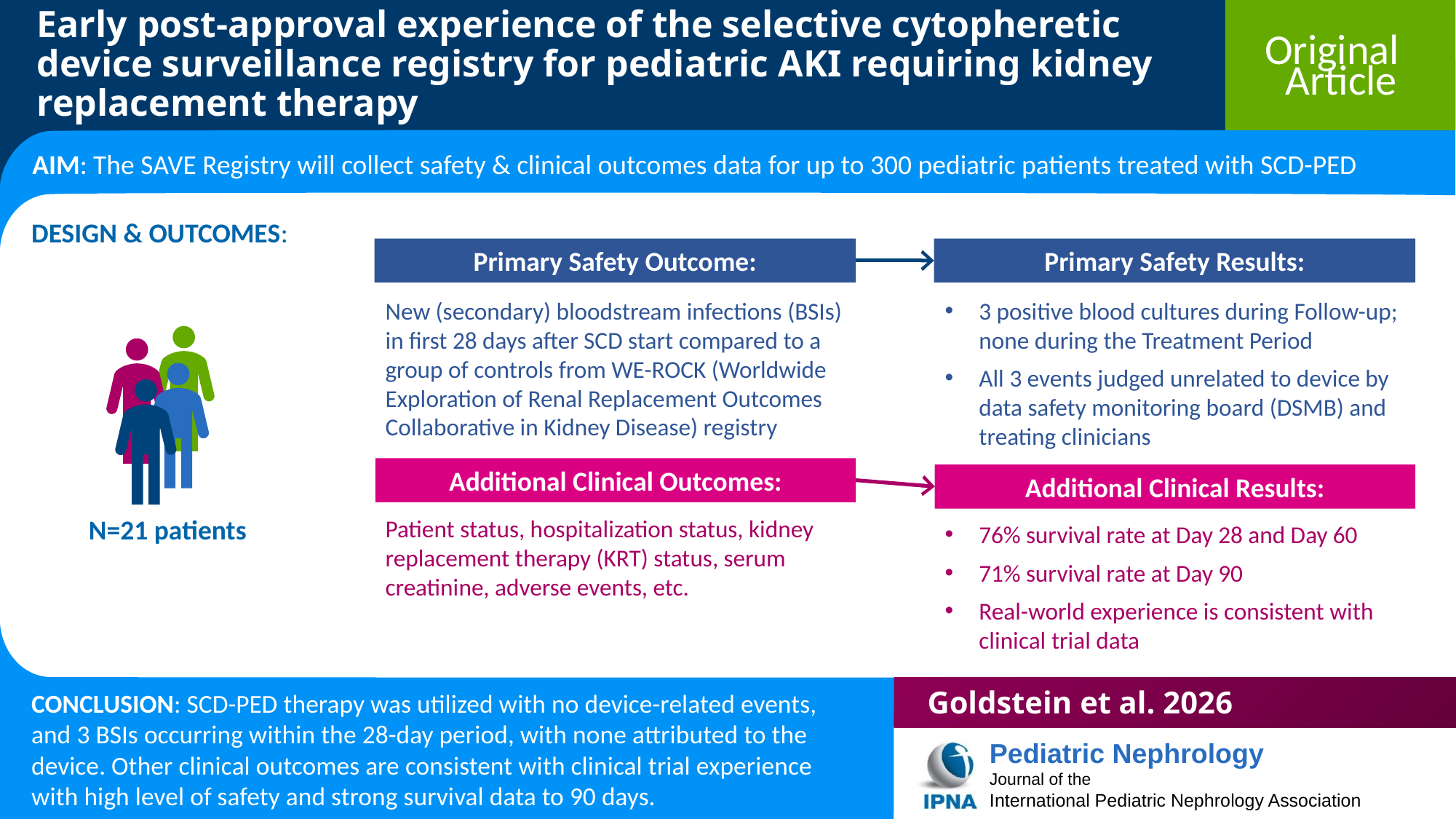

Early post-approval experience of the selective cytopheretic device surveillance registry for pediatric AKI requiring kidney replacement therapy
AIM: The SAVE Registry will collect safety & clinical outcomes data for up to 300 pediatric patients treated with SCD-PED
DESIGN & OUTCOMES:
Primary Safety Outcome:
New (secondary) bloodstream infections (BSIs) in first 28 days after SCD start compared to a group of controls from WE-ROCK (Worldwide Exploration of Renal Replacement Outcomes Collaborative in Kidney Disease) registry
Primary Safety Results:
3 positive blood cultures during Follow-up; none during the Treatment Period
All 3 events judged unrelated to device by data safety monitoring board (DSMB) and treating clinicians
N=21 patients
Additional Clinical Outcomes:
Patient status, hospitalization status, kidney replacement therapy (KRT) status, serum creatinine, adverse events, etc.
Additional Clinical Results:
76% survival rate at Day 28 and Day 60
71% survival rate at Day 90
Real-world experience is consistent with clinical trial data
Goldstein et al. 2026
CONCLUSION: SCD-PED therapy was utilized with no device-related events, and 3 BSIs occurring within the 28-day period, with none attributed to the device. Other clinical outcomes are consistent with clinical trial experience with high level of safety and strong survival data to 90 days.
