# Supplementary material for: Early post-approval experience of the selective cytopheretic device surveillance registry for pediatric AKI requiring kidney replacement therapy
Source: Pediatr Nephrol. 2026 Feb 6;41(7):2205–12. doi: 10.1007/s00467-026-07181-1 (PMC13197363; doi:10.1007/s00467-026-07181-1)
Supplement: Supplementary file 2 — (DOCX 41.5 KB) [file 467_2026_7181_MOESM2_ESM.docx]

**Supplemental Table 1: Hospital Diagnosis and Select Patient Characteristics**

| **Patient** | **Gender**  **Age (y)** | **Height (cm)**  **Weight (kg)** | **Primary ICU Diagnosis**  *Initial Hospital Diagnoses* | **RRT prior to SCD** | **ECMO** | **ICU>CKRT (d)**  **CKRT>SCD (d)** | **# of SCD**  **Courses**  **DoT (d)** | **PRISMIII**  **PELOD2** | **Eligible for Previous**  **SCD Trials** |
| --- | --- | --- | --- | --- | --- | --- | --- | --- | --- |
| 1 | M  14.7 | 163.5  105 | **Acute pancreatitis**  *[Data missing]* |  | N | 21  0** | 3  33 | 17  6 | No |
| 2 | M  16.9 | 164  94.4 | **Coagulopathy requiring resuscitation**  *Gastric perforation with dehiscence* |  | N | 1  5 | 1  22 | 22  7 | No |
| 3 | F  5.1 | 113  16.5 | **Acute liver failure**  *[Data missing]* |  | N | 12  0** | 1  5 | 10  9 |  |
| 4 | F  13.6 | NA | **[Data missing]**  *Severe lung disease due to influenza and MRSA pneumonia* |  | N | 0  1 | 1  11 | 16  5 |  |
| 5 | F  9.15 | 116  22.1 | **Septic shock**  *[Data missing]* |  | N | 0  1 | 1  2 | 16  9 |  |
| 6 | F  19.3 | NA | **[Data missing]**  *Viral acute gastroenteritis* |  | N | 0  2 | 1  2 | 31  6 |  |
| 7 | F  4.9 | 111.8  30.9 | **Respiratory failure**  *Neuroblastoma* |  | N | 4  0 (9.1 hr) | 1  2 | 5  12 | No |
| 8* | F  23.7 | NA | **[Data missing]**  *Diastolic heart failure* |  | Y | 274  2 | 1  1 | 2  15 |  |
| 9 | M  17.4 | 45.1 | **Septic shock and respiratory failure**  *Abdominal pain* |  | N | 4  4 | 1  5 | 14  10 |  |
| 10 | M  7 | 15.5 | **Heart failure**  *[Data missing]* |  | Y | 143  3 | 1  2 | 3  9 |  |
| 11 | M  16.8 | 73 | **Shock**  *Shock* |  | N | 0  1 | 1  10 | 29  11 |  |
| 12* | F  1.1 | 9 | **Heart failure**  *Dilated cardiomyopathy* | Yes | N | 44  97 | 1  10 | 8  8 | No |
| 13 | F  6.2 | 21.5 | **Respiratory failure**  *Wilms Tumor patient intubated* |  | Y | 0  4 | 1  5 | 29  14 | No |
| 14 | M  5.2 | 19.8 | **Viral pneumonia vs CLABSI**  *Respiratory distress,*  *CKD Stage 5 s/p kidney transplant* | Yes | N | 0  3 | 1  8 | 9  3 | No |
| 15 | M  1.8 | 13.6 | **Respiratory failure, Strep pneumonia**  *[Data missing]* |  | Y | 10  3 | 1  3 | 26  7 |  |
| 16 | F  5.3 | 11.2 | **Hypoxic respiratory failure d/t parainfluenza**  *Hypoxia, septic shock* |  | N | 2  0** | 1  5 | 0  10 |  |
| 17 | M  12.8 | 38.2 | **Status post kidney transplant**  *Admitted for kidney transplant* | Yes | Y | 4  0 (17.4 hr) | 1  1 | 7  3 | No |
| 18 | F  18.8 | 52.2 | **SLE flare with pulmonary hemorrhage and mucormycosis**  *SLE flare w/complexities* |  | Prior to SCD | 18  0 (19.7 hr) | 1 7 | 15  15 | No |
| 19 | M  8 | 19.6 | **Fluid-responsive shock**  *Acute hypoxemic respiratory failure* | Yes | - | 74  41 | 1  6 | 10  9 | No |
| 20 | M  5.6 | 17.1 | **Post-surgical congenital heart disease; left-sided unifocalization**  *Unifocalization of the left sided major aortopulmonary collateral arteries, banding of the right pulmonary artery* |  | Prior to SCD | 1  33 | 1  6 | 15  6 |  |
| 21^1^ | M  10 | NA | **Neutropenic septic shock with MOF and DIC**  *Abdominal mass* |  | N | 1  2 | 2  11 | 22  12 | No |
| CLABSI: central line-associated bloodstream infection; DIC: disseminated intravascular coagulation; DoT: duration of therapy; ECMO: extracorporeal membrane oxygenation; CKD: chronic kidney disease; CKRT: continuous kidney replacement therapy; MOF: multi-organ failure; MRSA: methicillin-resistant *Staphylococcus aureus*; NA: not available; PELOD-2: pediatric logistic organ dysfunction score; PRISM III: pediatric risk of mortality score; SCD: selective cytopheretic device; SLE: systemic lupus erythematosus; s/p: status post; *initially treated under emergency use and subsequently added to registry; **patients were concurrently initiated with CKRT and SCD. | | | | | | | | | |

**References**:

1. Humes HD, Luckritz K, Gorga S*, et al.* Management dilemma in choosing evolving treatments in neutropenic septic shock. *Pediatr Nephrol* 2025.
