# Supplementary material for: Early post-approval experience of the selective cytopheretic device surveillance registry for pediatric AKI requiring kidney replacement therapy
Source: Pediatr Nephrol. 2026 Feb 6;41(7):2205–12. doi: 10.1007/s00467-026-07181-1 (PMC13197363; doi:10.1007/s00467-026-07181-1)
Supplement: Supplementary file 3 — (DOCX 24.0 KB) [file 467_2026_7181_MOESM3_ESM.docx]

**Supplemental Table 2: Additional Details on Patients with History of CKD Stage 5 and/or Recent Transplant.**

Four patients with a documented history chronic kidney disease (CKD) stage 5 and/or prior renal transplantation were initiated on SCD-PED therapy following an acute deterioration in renal function. The decision to commence therapy was made by the respective treating clinicians, based on their clinical judgment that modulation of the patients’ inflammatory burden could offer therapeutic benefit.

| **Patient** | **Primary ICU Diagnosis**  *Initial Hospital Diagnoses* | **RRT prior**  **to SCD** | **Additional Details** |
| --- | --- | --- | --- |
| 12 | **Heart failure**  Dilated cardiomyopathy | Yes | History of anuric renal failure with dialysis dependence since 2024 and was ongoing at hospital admission. Patient was treated with SCD-PED initially under emergency use and subsequently allowed for inclusion in the registry by FDA. |
| 14 | **Viral pneumonia vs CLABSI**  *Respiratory distress,*  *CKD 5 s/p kidney transplant* | Yes | Patient with kidney transplant and mild CKD who developed acute infection that led to AKI requiring RRT. |
| 17 | **Status post kidney transplant**  *Admitted for kidney transplant* | Yes | Admitted to hospital for kidney transplant with history of CKD stage 5 since 2024. Had delayed graft function, followed by vasoplegic septic shock requiring ECMO and RRT. |
| 19 | **Fluid-responsive shock**  *Acute hypoxemic respiratory failure* | Yes | History of CKD Stage 5 (peritoneal dialysis in 2024) and was dialysis dependent before his illness and ICU presentation, which involved an acute worsening in renal status. |
| AKI: acute kidney injury; CKD: chronic kidney disease; CLABSI: central line-associated bloodstream infection; ECMO: extracorporeal membrane oxygenation; FDA: U.S. Food & Drug Administration; ICU: intensive care unit; RRT: renal replacement therapy; SCD-PED: selective cytopheretic device for pediatrics; s/p: status post | | | |
