# Supplementary material for: Early post-approval experience of the selective cytopheretic device surveillance registry for pediatric AKI requiring kidney replacement therapy
Source: Pediatr Nephrol. 2026 Feb 6;41(7):2205–12. doi: 10.1007/s00467-026-07181-1 (PMC13197363; doi:10.1007/s00467-026-07181-1)
Supplement: Supplementary file 4 — (DOCX 36.4 KB) [file 467_2026_7181_MOESM4_ESM.docx]

**Supplemental Table 3: Individual Patient Outcomes at Day 28, 60 and 90**

| **Patient** | **Alive Day 28**  **(Y/N)** | **KRT Independent**  **at Day 28**  **(non-CKD Stage Survivors)**  **(Y/N)** | **Alive Day 60**  **(Y/N)** | **Alive Day 90**  **(Y/N)** | **KRT Independent**  **at Day 90**  **(non-CKD Stage 5 Survivors)**  **(Y/N)** |
| --- | --- | --- | --- | --- | --- |
| 1 | Y | N | Y | Y | N |
| 2 | Y | N | Y | Y | N |
| 3 | Y | Y | Y | Y | Y |
| 4 | Y | Y | Y | Y | Y |
| 5 | Y | Y | Y | Y | Y |
| 6 | Y | Y | Y | Y | Y |
| 7 | N | N/A | N | N | N/A |
| 8 | N |  | N | N |  |
| 9 | N |  | N | N |  |
| 10 | N |  | N | N |  |
| 11 | Y | Y | Y | Y | Y |
| 12 | Y | N | Y | N | N/A |
| 13 | Y | N | Y | Y | Y |
| 14 | Y | N | Y | Y | N |
| 15 | Y | Y | Y | Y | Y |
| 16 | Y | Y | Y | Y | Y |
| 17 | Y | N | Y | Y | N |
| 18 | N | N/A | N | N | N/A |
| 19 | Y | N | Y | Y | N |
| 20 | Y | Y | Y | Y | Y |
| 21^1^ | Y | Y | Y | Y | Y |
| **Totals** | **Survival**  **Day 28** | **RRT Independent**  **Day 28** | **Survival**  **Day 60** | **Survival**  **Day 90** | **RRT Independent**  **Day 90** |
| **N**  **(%)** | **16 of 21**  **(76%)** | **9 of 12**  **(75%)** | **16 of 21**  **(76%)** | **15 of 21**  **(71%)** | **10 of 12**  **(83%)** |
| Red shading: CKD Stage 5/dialysis-dependent at baseline; Gray shading: subject died; CKD: chronic kidney disease; KRT: kidney replacement therapy | | | | | |

1. Humes HD, Luckritz K, Gorga S*, et al.* Management dilemma in choosing evolving treatments in neutropenic septic shock. *Pediatr Nephrol* 2025.
