# Supplementary material for: Early post-approval experience of the selective cytopheretic device surveillance registry for pediatric AKI requiring kidney replacement therapy
Source: Pediatr Nephrol. 2026 Feb 6;41(7):2205–12. doi: 10.1007/s00467-026-07181-1 (PMC13197363; doi:10.1007/s00467-026-07181-1)
Supplement: Supplementary file 5 — (DOCX 23.9 KB) [file 467_2026_7181_MOESM5_ESM.docx]

**Supplemental Table 4: Additional Information on Positive Bloodstream Infections**

| **Positive Blood Culture:** | **Details:** |
| --- | --- |
| Ampicillin-resistant *Klebsiella* | This patient was a 1.1-year-old female with admitted to hospital with a congenital heart disease (dilated cardiomyopathy) and was awaiting a heart transplant.  She was in the ICU for 44 days, followed by treatment on CKRT for an additional 97 days before getting initiated and treated with the SCD-PED for 10 days.  The patient died 51 days after end of therapy due to their underlying illness. On the day of the index event (6 days post-end of treatment; ~17 days post-initiation with SCD-PED) and thus within the 28-day capture window for the primary endpoint of BSIs:   - 01/17 10:14 AM (central line specimen source) – Klebsiella resistant to ampicillin   The database entry on the following day (drawn approximately 32 hours after the previous culture was drawn) stated:   - 01/18 6:01 PM (central line specimen source) – negative   An additional subsequent culture the next day was also negative:   - 01/19 2:01 PM (central line specimen source) – negative   Upon further adjudication, the positive blood culture was deemed not associated with SCD-PED therapy by the DMSB and treating clinician.  It is unknown whether the patient was positive for the full time between the positive and negative cultures or whether the entry may have been made in error, and it is impossible to rule out contamination. |
| *Enterococcus faecalis* | Occurred 7 days after end of SCD-PED treatment. Adjudicated as unrelated to SCD-PED therapy by treating clinician and DSMB. |
| *Enterobacter* | Occurred 22 days after end of SCD-PED treatment. Adjudicated as unrelated to SCD-PED therapy by treating clinician and DSMB. |
| CKRT: continuous kidney replacement therapy; DSMB: data safety monitoring board; ICU: intensive care unit; SCD-PED: selective cytopheretic device for pediatrics | |
